# The need for hands-on training and supervision for entry-level physicians in a country with low surgical staffing density: a nationwide survey in Ghana

**DOI:** 10.1186/s12909-023-04880-3

**Published:** 2023-11-29

**Authors:** Mee Joo Kang, Reuben Kwesi Sakyi Ngissah, Alexis Dun Bo-Ib Buunaaim, Richard Baidoo, Francis Odei-Ansong, Theodore Wordui, Ernest Kwame Adjepong-Tandoh, Paa Kwesi Baidoo, James Emmanuel Kwegyir Aggrey-Orleans

**Affiliations:** 1 Department of Surgery, Greater Accra Regional Hospital, P.O.Box 473, Accra, Republic of Ghana; 2 Department of Surgery, National Cancer Center, Goyang, Republic of Korea; 3 Department of Surgery, Tamale Teaching Hospital, Tamale, Republic of Ghana; 4 Department of Surgery, Cape Coast Teaching Hospital, Cape Coast, Republic of Ghana; 5 Department of Surgery, Tema General Hospital, Tema, Republic of Ghana; 6 Department of Surgery, Korle-Bu Teaching Hospital, Accra, Republic of Ghana; 7 Department of Surgery, Komfo-Anokye Teaching Hospital, Kumasi, Republic of Ghana; 8 Department of Surgery, 37 Military Hospital, Accra, Republic of Ghana

**Keywords:** Surgery, Education, Internship, Self-confidence, Clinical supervision

## Abstract

**Background:**

Despite the largely unmet need, relatively few medical school graduates enrol in surgical residency and fewer surgical specialists work rurally in low- and middle-income countries. Surgical housemanship is the only formal training for medical graduates who will become the main surgical care providers in underserved areas. This study aimed to evaluate Ghanaian surgical housemanship (internship) and its impact on independent medical practice.

**Methods:**

A nationwide questionnaire survey of surgical trainees from seven teaching or regional-level hospitals ascertained the experience and self-confidence levels for 35 training objectives set by the Medical and Dental Council of Ghana, and suggestions to improve surgical training quality.

**Results:**

Of 310 respondents, 59.7% experienced ≤ 10 cases for each topic, and 24.8% reported self-confidence as ≤ 2 points (out of 5). More than 90% of respondents experienced ≤ 10 cases for gastric, colorectal and liver cancer management. Teaching hospital trainees had lower proportions of those experiencing > 10 cases (36.6% versus 43.7%) and reporting self-confidence ≥ 4 (46.5% versus 55.8%), respectively, compared with those from regional/other-level hospitals. 40% of respondents were not confident about their surgical skills, and 70.5% requested better-supervised and practical surgical skills training. The proportion of respondents who reported limited supervision was higher among those from teaching hospitals, reported self-confidence scores < 4, and experienced ≤ 10 cases for each topic. 67% of respondents were satisfied with their surgical housemanship and 75.8% perceived surgical rotation as relevant to their future work.

**Conclusions:**

Most surgical trainees are concerned about their surgical skills. A structured curriculum with specific goals and better-supervised surgical skills training should be established. Inclusion of regional/other-level hospitals in surgical training may reduce the supervisory burden in teaching hospitals.

**Supplementary Information:**

The online version contains supplementary material available at 10.1186/s12909-023-04880-3.

## Introduction

Although surgically treatable conditions account for 28–32% of the overall global burden of disease, many low- and middle-income countries (LMICs) have limited access to basic surgical care and a shortage of surgical, anaesthetic, and obstetric providers [1]. Lancet Commission on Global Surgery estimated that to achieve the goal of 80% global access to safe surgery, obstetric care, trauma, and anesthesia by 2030 [2], it necessitates a surgical and anesthetic workforce density, with a minimum requirement of 20 professionals per 100,000 population [1]. Since the inception of WHA68.15 [3], there have been gradual improvements in surgical and anesthesia care; however, comprehensive data in this regard is somewhat limited. Regrettably, as of the present day, many LMICs fall significantly short of this critical benchmark [4]. To address this disparity and strengthen surgical workforce density in these regions, there is an urgent need for an expansion of the capacity for training surgical specialists.

West African countries have had well-established postgraduate surgical training programmes since 1960, when the West African College of Surgeons (WACS) was founded. Along with the WACS, the Ghana College of Physicians and Surgeons (GCPS) has provided a standard format for surgical training programmes and diplomas since 2003 [5]. The annual operation rate in Ghana is 869 per 100,000 people, which is less than 20% of the surgery rate benchmark set by the Lancet Commission of Global Surgery (5,000 per 100,000 people) [6]. Despite the largely unmet need, relatively few medical school graduates enrol in surgical residency and fewer surgical specialists work rurally [7, 8]. Between 2014 and 2015, district-level hospitals performed 62% of all operations in Ghana; however, 54% of the hospitals did not have fully trained surgeons [6]. Medical officers (MOs) who have completed 2 years of housemanship (i.e. internship) are the main surgical care providers in Ghanaian district hospitals [9]. Therefore, evaluation of the status quo of the house officer (HO) training programme and its impact on the practice of MOs is necessary to improve the surgical training quality during housemanship, which is the only and final opportunity for formal surgical training.

In 2019, preliminary results from a study in a regional hospital in Ghana revealed that lack of experience and self-confidence in cancer management and surgical skills, and limited supervision were the major drawbacks of surgical housemanship [7]. However, the study was not representative of the overall situation in Ghana because it investigated only one regional hospital located in the capital city.

Therefore, this study expanded the coverage to a national scale, including major teaching and regional-level hospitals designated for HO training. In addition, the questionnaire survey was extended to MOs working at various hospital levels to evaluate the impact of surgical housemanship on their practice. Through this questionnaire, the opinions of frontline surgical care providers with the best understanding of challenges on the ground were investigated. This study reports the volume of experience and self-confidence in surgical conditions or procedures, drawbacks of surgical housemanship, suggestions for improvement of surgical training quality, and major obstacles faced by MOs on the ground.

## Methods

During a 2-year housemanship, a 6-month surgical rotation is compulsory. The principles of the housemanship programme in Ghana have been described in our previous study [7]. This study is a national scale-up of our previous study, expanding its scope to both HOs and MOs. The 35 conditions or procedures established as teaching objectives for surgical HOs by the Medical and Dental Council (MDC) of Ghana were assessed [10]. Since most hospitals in Ghana do not have specialists in each field, the 35 teaching objectives include medical topics needed to manage surgical patients. This cross-sectional nationwide study was approved by the Ethics Review Committee of Ghana Health Service and in accord with the ethical standards of the Helsinki Declaration of 1975.

### Study site

Currently, there are five teaching hospitals in Ghana. Four of five teaching hospitals and three regional-level hospitals designated for HO training participated in this study. The hospital locations were as follows: the capital city Accra, Greater Accra Region (Greater Accra Regional Hospital, Korle-Bu Teaching Hospital, and 37 Military Hospital); Tema, Greater Accra Region (Tema General Hospital); Kumasi, Ashanti Region (Komfo-Anokye Teaching Hospital); Cape Coast, Central Region (Cape Coast Teaching Hospital); and Tamale, Northern Region (Tamale Teaching Hospital).

### Data collection

From August to September 2021, a self-administered electronic questionnaire was sent to 500 HOs who had completed surgical rotation and MOs who had worked or were currently working in the seven participating hospitals. The survey link was sent via text message and the contact information of the HOs and MOs was obtained from each participating hospital. The internal consistency and construct validity of the questionnaire were validated in a pilot study [7]. Questionnaire responses were voluntary and anonymous. The number of cases experienced and self-confidence in the 35 conditions or procedures established by the MDC of Ghana, satisfaction level with the surgical rotation, and relevance of surgical rotation to their future work were rated on a 5-point Likert-type scale (Table S1) [7]. The 35 conditions or procedures were aggregated into the following domains: general medical management (acute renal failure, anaemia, blood transfusion, diabetes and its complications, fluid and electrolyte therapy, nutrition in surgery, and shock), general surgical management (pre- and post-operative care, preparation for and test for fitness for surgery, surgical infections, and wounds), hernia (inguinoscrotal hernia), trauma and injuries (application of Plaster of Paris [POP], chest injuries, fracture management, head injuries, and the injured patient), urology (haematuria, retention of urine, including benign prostate hyperplasia/prostate cancer and urethral stricture), surgical abdomen (acute abdomen, appendicitis, gastrointestinal bleeding, intestinal obstruction, peptic ulcer disease and complications, and typhoid), cancer (breast, colorectal, gastric, and liver cancers, cancer chemotherapy/cancer therapy, and portal hypertension), and others (burns, hand infections, jaundice, and peripheral vascular disease). The criteria of using 10 cases as a cutoff for analysis were established based on the average caseload in clinical practice at a regional hospital in Ghana. The number of cases experienced was recorded in the questionnaire because most HOs fill their logbooks towards the end of the year, with a higher risk of recall bias and inaccuracy. Regarding the levels of satisfaction and relevance, ≥ 4 points were considered satisfactory and relevant. Although self-confidence does not directly reflect the level of surgical competency [11], a certain level of self-confidence is required for an independent doctor working at district- or sub-district-level hospitals. In this study, self-confidence and satisfaction levels were used as proxy measures of experience and surgical competency obtained during surgical rotation [7, 12].

### Data analysis

Statistical analysis was conducted using STATA version 15.1 (Stata Corp, Texas, USA). The aggregated scores for the number of cases experienced and self-confidence were derived from Likert-type scale scores in all 35 sub-domains. Summary measures are presented as numbers and percentages. Continuous variables were analysed using the Wilcoxon rank-sum test, and nominal variables were analysed using the chi-square test or Fisher’s exact test and Spearman’s correlation test. Two-sided *p* values < 0.05 were considered statistically significant. Thematic analysis was performed to analyse the results of the open-ended questions on the evaluation forms.

## Results

### Respondents’ characteristics

The overall response rate was 62% (310 of 500). Among the 310 respondents, 45.2% (*n* = 140) were HOs, 40.3% (*n* = 125) were MOs, and 14.5% (*n* = 45) had completed housemanship but were yet to be posted as MOs. 47% of respondents (*n* = 145) had their surgical housemanship in teaching hospitals and 53% were trained in regional or district-level hospitals. 55% (*n* = 170) of respondents were men and 45% (*n* = 140) were women. 75% (*n* = 234), 20% (*n* = 63), and 4% (*n* = 13) of respondents were in their 20s, 30s, and 40s or older, respectively.

### Self-reported volume of cases experienced during surgical rotation

**Fig. 1:**
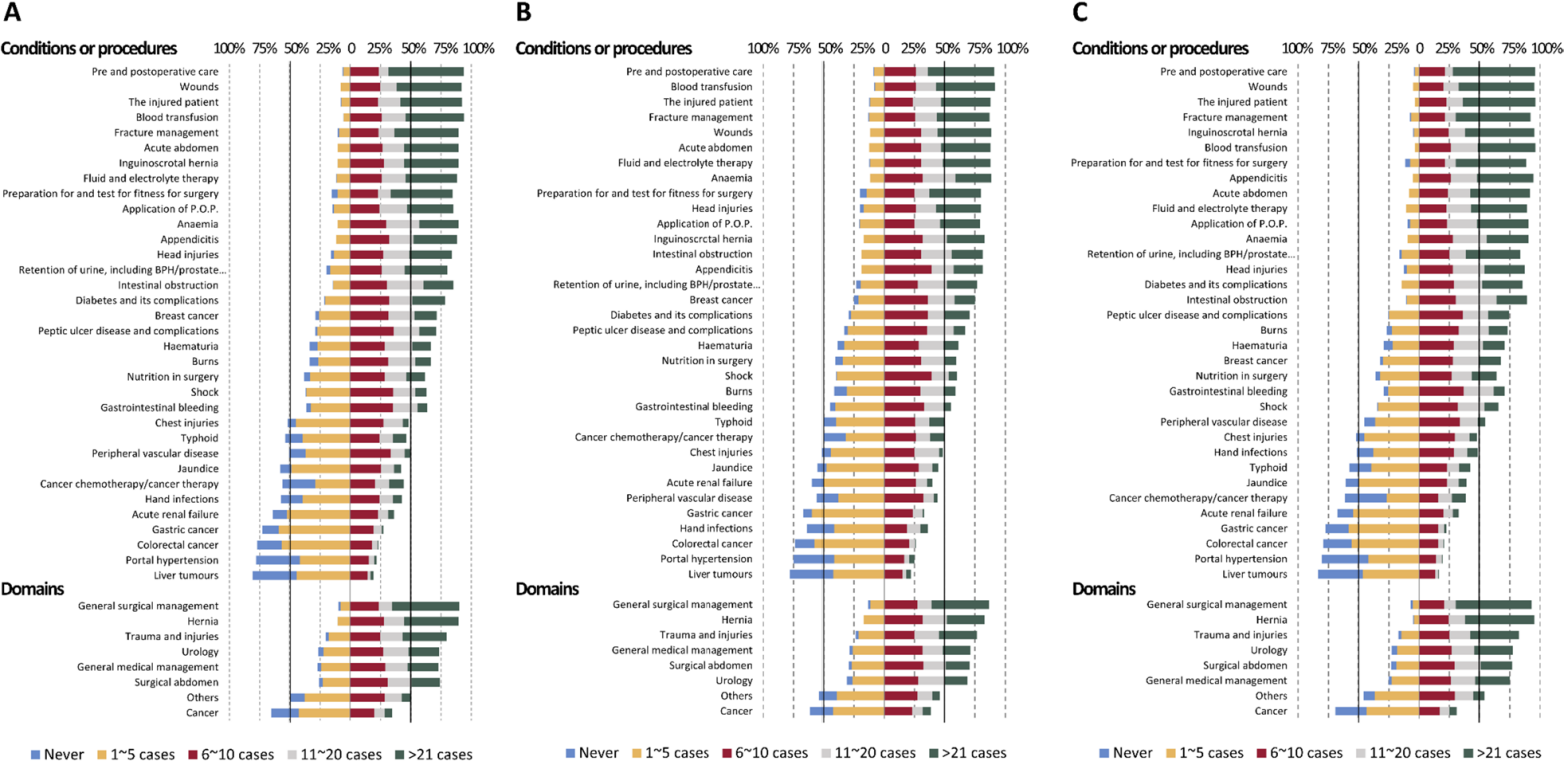
Self-reported number of cases experienced: **A** Overall respondents; **B** Respondents trained at teaching hospitals; **C** Respondents trained at regional or other-level hospitals

The proportion of those experienced ≤ 10 cases for each condition or procedure during the 6-month surgical rotation was 59.7%. Teaching hospitals had a higher proportion of respondents experiencing ≤ 10 cases for each condition or procedure than did regional/other-level hospitals (63.4% vs. 56.3%, *p* < 0.001). The proportions of the volume of experience for each condition or procedure are shown in Fig. 1. Comparing domains, the volume of experience was high in general surgical and hernia managements, but low in cancer management. Exposure to trauma and injuries, urology, general medical management, and surgical abdomen had comparable levels of experience. The trend was comparable between those who were trained in teaching and regional/other-level hospitals (Fig. 1). To investigate the conditions or procedures for which experience was limited, the proportions of those who had experienced ≤ 10 cases are shown in Table 1. More than 80% of the respondents experienced ≤ 10 cases of the following 8 conditions or procedures: hand infections, jaundice, peripheral vascular disease, acute renal failure, gastric cancer, portal hypertension, colorectal cancer and liver tumours. More than 90% of all respondents had experience with ≤ 10 cases of gastric, colorectal, and liver cancers; in contrast, a higher volume of breast cancer cases was experienced. Comparing the volume of experience according to the training hospitals, those who were trained in teaching hospitals had less experience with wounds, inguinoscrotal hernia, POP application, appendicitis, diabetes and its complications, burns, gastrointestinal bleeding, shock, and peripheral vascular disease compared with those from regional/other-level hospitals. The volume of experience with breast, gastric, colorectal, and liver cancers was comparable between the two groups (Table 1).

**Table 1:** Proportions of house and medical officers who experienced 10 or fewer cases during their surgical rotation

Conditions or procedures	Experienced ≤ 10 cases			
	Overall (*n* = 310)	Trained at teaching hospitals (*n* = 145)	Trained at regional/other-level hospitals (*n* = 165)	*p*-value (Teaching vs. regional/other-level hospitals)
Pre and postoperative care	93 (30.0%)	51 (35.2%)	42 (25.5%)	0.062
The injured patient	95 (30.6%)	52 (35.9%)	43 (26.1%)	0.062
Blood transfusion	99 (31.9%)	50 (34.5%)	49 (29.7%)	0.367
Wounds	102 (32.9%)	61 (42.1%)	41 (24.8%)	0.001
Fracture management	104 (33.5%)	56 (38.6%)	48 (29.1%)	0.076
Acute abdomen	115 (37.1%)	62 (42.8%)	53 (32.1%)	0.053
Fluid and electrolyte therapy	117 (37.7%)	62 (42.8%)	55 (33.3%)	0.088
Preparation for and test for fitness for surgery	119 (38.4%)	65 (44.8%)	54 (32.7%)	0.029
Inguinoscrotal hernia	119 (38.4%)	71 (49.0%)	48 (29.1%)	< 0.001
Application of Plaster of Paris	120 (38.7%)	66 (45.5%)	54 (32.7%)	0.021
Anaemia	125 (40.3%)	63 (43.4%)	62 (37.6%)	0.293
Head injuries	134 (43.2%)	67 (46.2%)	67 (40.6%)	0.321
Appendicitis	136 (43.9%)	84 (57.9%)	52 (31.5%)	< 0.001
Intestinal obstruction	139 (44.8%)	71 (49.0%)	68 (41.2%)	0.171
Retention of urine, including BPH/prostate cancer and urethral stricture	142 (45.8%)	74 (51.0%)	68 (41.2%)	0.083
Diabetes and its complications	167 (53.9%)	95 (65.5%)	72 (43.6%)	< 0.001
Breast cancer	187 (60.3%)	88 (60.7%)	99 (60.0%)	0.901
Haematuria	192 (61.9%)	97 (66.9%)	95 (57.6%)	0.092
Burns	201 (64.8%)	103 (71.0%)	98 (59.4%)	0.032
Peptic ulcer disease and complications	201 (64.8%)	99 (68.3%)	102 (61.8%)	0.235
Nutrition in surgery	206 (66.5%)	103 (71.0%)	103 (62.4%)	0.109
Gastrointestinal bleeding	222 (71.6%)	113 (77.9%)	109 (66.1%)	0.021
Shock	225 (72.6%)	115 (79.3%)	110 (66.7%)	0.013
Cancer chemotherapy/cancer therapy	237 (76.5%)	110 (75.9%)	127 (77.0%)	0.819
Typhoid	242 (78.1%)	109 (75.2%)	133 (80.6%)	0.249
Chest injuries	246 (79.4%)	111 (76.6%)	135 (81.8%)	0.253
Hand infections	253 (81.6%)	120 (82.8%)	133 (80.6%)	0.625
Jaundice	259 (83.5%)	121 (83.4%)	138 (83.6%)	0.964
Peripheral vascular disease	259 (83.5%)	128 (88.3%)	131 (79.4%)	0.035
Acute renal failure	269 (86.8%)	125 (86.2%)	144 (87.3%)	0.782
Gastric cancer	285 (91.9%)	131 (90.3%)	154 (93.3%)	0.335
Portal hypertension	289 (93.2%)	133 (91.7%)	156 (94.5%)	0.324
Colorectal cancer	294 (94.8%)	137 (94.5%)	157 (95.2%)	0.791
Liver tumours	295 (95.2%)	135 (93.1%)	160 (97.0%)	0.113

Values are n (%)

### Self-reported confidence after completing surgical rotation

The proportion of those with a self-reported confidence score of ≤ 2 (out of 5) was 24.8% overall. The proportion of those reporting self-confidence scores ≥ 4 was higher among those from regional/other-level hospitals than teaching hospitals (55.8% [regional/other-level hospitals] vs. 46.5% [teaching hospitals], *p* < 0.001).

**Fig. 2:**
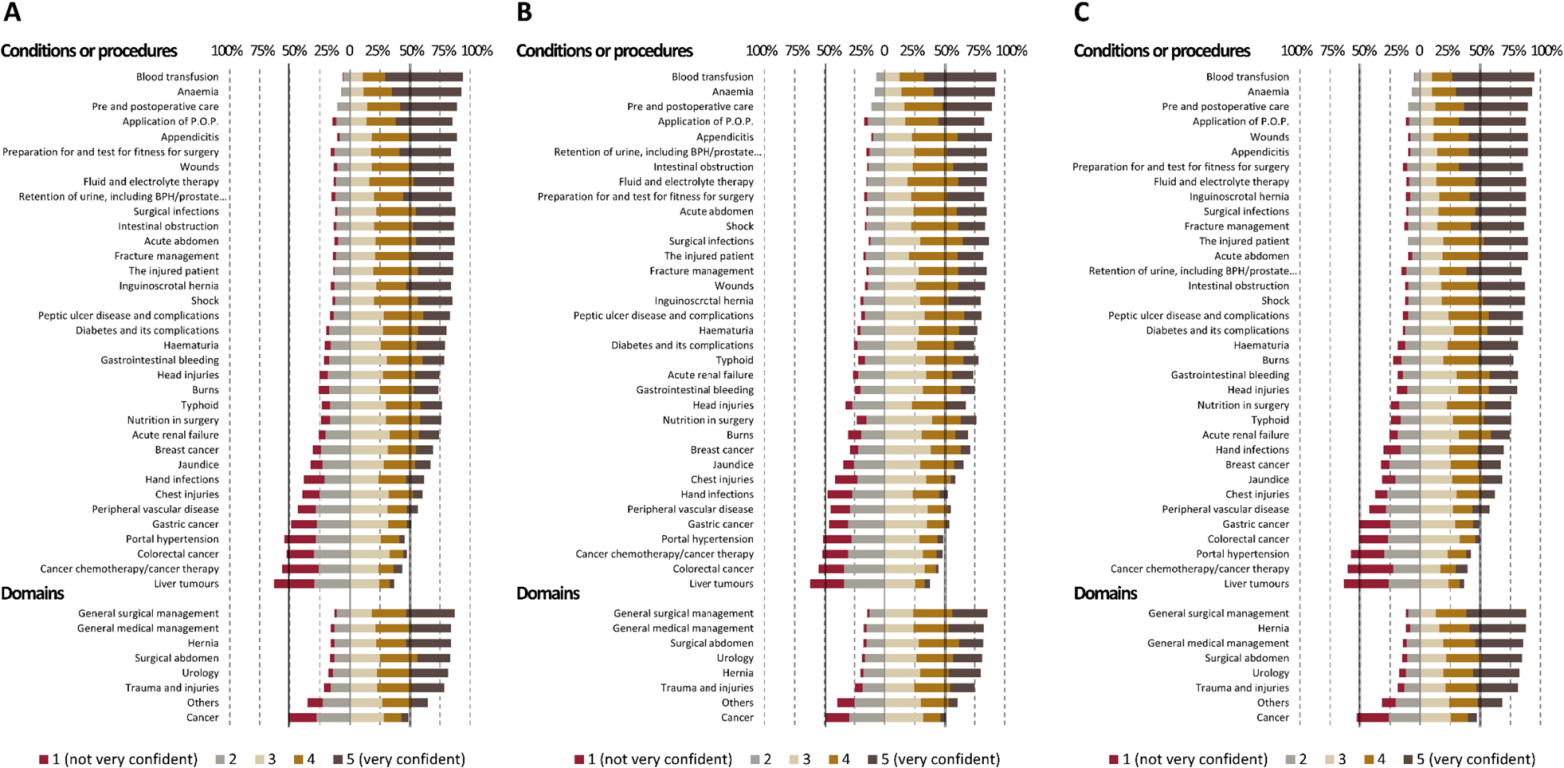
Self-reported confidence score: **A** Overall respondents; **B** Respondents trained at teaching hospitals; **C** Respondents trained at regional or other-level hospitals

The proportion of self-reported confidence levels for each condition or procedure is presented in Fig. 2. Self-confidence levels were higher for the general surgical and medical managements, hernia, surgical abdomen, and urology domains, while they were lower for cancer management. The trend was comparable between those who were trained in teaching and regional/other-level hospitals (Fig. 2).

The proportion of those with a self-confidence score ≤ 2 is shown in Table 2. More than 50% of respondents had self-confidence scores of ≤ 2 in four conditions or procedures: portal hypertension, colorectal and liver cancers, and cancer chemotherapy. Compared with other conditions, these had a higher proportion of respondents who experienced ≤ 10 cases during surgical rotation (liver tumours, 95.2%; portal hypertension, 93.2%; colorectal cancer, 94.8%; cancer chemotherapy/cancer therapy, 76.5%). Those who were trained in teaching hospitals had lower self-confidence levels for diabetes and its complications, head injuries, and hand infections than did those from regional/other-level hospitals (Table 2).

**Table 2:** Proportions of house and medical officers who reported self-confidence of 1 or 2 out of 5 points

Conditions or procedures	Confidence ≤ 2 points			
	Overall (*n* = 310)	Trained at teaching hospitals (*n* = 145)	Trained at regional/other-level hospitals (*n* = 165)	*p*-value (Teaching vs. regional/other-level hospitals)
Blood transfusion	18 (5.8%)	10 (6.9%)	8 (4.8%)	0.474
Anaemia	23 (7.4%)	12 (8.3%)	11 (6.7%)	0.666
Pre and postoperative care	33 (10.6%)	16 (11.0%)	17 (10.3%)	0.856
Application of Plaster of Paris	45 (14.5%)	25 (17.2%)	20 (12.1%)	0.258
Appendicitis	33 (10.6%)	16 (11.0%)	17 (10.3%)	0.856
Preparation for and test for fitness for surgery	49 (15.8%)	25 (17.2%)	24 (14.5%)	0.536
Wounds	41 (13.2%)	24 (16.6%)	17 (10.3%)	0.130
Fluid and electrolyte therapy	41 (13.2%)	22 (15.2%)	19 (11.5%)	0.402
Retention of urine, including BPH/prostate cancer and urethral stricture	48 (15.5%)	22 (15.2%)	26 (15.8%)	> 0.999
Surgical infections	38 (12.3%)	19 (13.1%)	19 (11.5%)	0.730
Intestinal obstruction	42 (13.5%)	21 (14.5%)	21 (12.7%)	0.740
Acute abdomen	39 (12.6%)	22 (15.2%)	17 (10.3%)	0.231
Fracture management	44 (14.2%)	22 (15.2%)	22 (13.3%)	0.745
The injured patient	43 (13.9%)	26 (17.9%)	17 (10.3%)	0.069
Inguinoscrotal hernia	49 (15.8%)	29 (20.0%)	20 (12.1%)	0.063
Shock	45 (14.5%)	24 (16.6%)	21 (12.7%)	0.419
Peptic ulcer disease and complications	52 (16.8%)	28 (19.3%)	24 (14.5%)	0.288
Diabetes and its complications	61 (19.7%)	37 (25.5%)	24 (14.5%)	0.021
Haematuria	64 (20.6%)	33 (22.8%)	31 (18.8%)	0.402
Gastrointestinal bleeding	67 (21.6%)	36 (24.8%)	31 (18.8%)	0.215
Head injuries	79 (25.5%)	47 (32.4%)	32 (19.4%)	0.009
Burns	81 (26.1%)	44 (30.3%)	37 (22.4%)	0.122
Typhoid	72 (23.2%)	32 (22.1%)	40 (24.2%)	0.687
Nutrition in surgery	74 (23.9%)	34 (23.4%)	40 (24.2%)	0.894
Acute renal failure	80 (25.8%)	38 (26.2%)	42 (25.5%)	0.897
Breast cancer	96 (31.0%)	42 (29.0%)	54 (32.7%)	0.538
Jaundice	102 (32.9%)	50 (34.5%)	52 (31.5%)	0.629
Hand infections	119 (38.4%)	69 (47.6%)	50 (30.3%)	0.002
Chest injuries	122 (39.4%)	60 (41.4%)	62 (37.6%)	0.560
Peripheral vascular disease	135 (43.5%)	65 (44.8%)	70 (42.4%)	0.731
Gastric cancer	151 (48.7%)	67 (46.2%)	84 (50.9%)	0.427
Portal hypertension	169 (54.5%)	74 (51.0%)	95 (57.6%)	0.256
Colorectal cancer	163 (52.6%)	80 (55.2%)	83 (50.3%)	0.426
Cancer chemotherapy/cancer therapy	175 (56.5%)	75 (51.7%)	100 (60.6%)	0.136
Liver tumours	195 (62.9%)	90 (62.1%)	105 (63.6%)	0.814

Values are n (%)

The self-reported confidence scores positively correlated with the volume of experience (Spearman’s correlation coefficient 0.541, *p* < 0.001). Those who rated their confidence ≥ 4 points were more likely to experience > 10 cases (25.3% [confidence score ≥ 4] vs. 6.6% [confidence score < 4]; *p* < 0.001).

### Level of satisfaction and relevance to future work

Of all respondents, 66.8% (*n* = 207) were satisfied with their surgical rotation, and 75.8% (*n* = 235) perceived surgical rotation as relevant to their work after completing housemanship. Satisfaction with surgical rotation was significantly higher among respondents with confidence scores ≥ 4 (91.6% [confidence score ≥ 4] vs. 57.7% [confidence score < 4], *p* < 0.001), who were trained in regional/other-level hospitals (78.8% [regional/other-level hospitals] vs. 53.1% [teaching hospitals], *p* < 0.001) and perceived surgical rotation as relevant to their future work (81.7% [relevance score ≥ 4] vs. 20.0% [relevance score < 4], *p* < 0.001).

### Reasons for not feeling confident after surgical rotation

The areas in which respondents felt most confident after their surgical rotations were preoperative management, postoperative management, and knowledge of basic principles (Fig. S1). More than 40% (*n* = 125) of respondents did not feel confident with their surgical skills. The reasons for this lack of confidence included limitations in number of patients (76.5%), resources and infrastructure (45.5%), and supervision (38.1%, Table S2). The proportion of respondents who reported limited supervision were higher among those trained in teaching hospitals (44.8% [teaching hospitals] vs. 32.1% [regional/other-level hospitals], *p* = 0.022), reported self-confidence scores < 4 (42.7% [confidence score < 4] vs. 25.3% [confidence score ≥ 4], *p* = 0.005), and experienced ≤ 10 cases for each condition or procedure (40.5% [experienced ≤ 10 cases] vs. 19.4% [experienced > 10 cases], *p* = 0.014). Those who are currently working as MOs in non-surgical disciplines reflected that they were disinterested in surgical conditions (12.3% [non-surgical disciplines] vs. 2.9% [surgical disciplines], *p* = 0.015). The proportion of respondents who reported limited resources and infrastructure as a reason for lack of confidence was comparable among the hospitals where they were trained, number of cases experienced, and confidence levels. A multivariate analysis revealed that training in regional/other-level hospitals (odds ratio [OR], 2.550; 95% confidence interval [CI], 1.437–4.523; *p* = 0.001) and experiencing > 10 cases for each condition or procedure (OR, 5.557; 95% CI, 2.615–11.810; *p* < 0.001) were independent factors related to a confidence score ≥ 4.

### Suggestions to improve the quality of surgical rotations

The overall response rate for an open-ended question requesting suggestions to improve the surgical rotation quality (Table 3) was 48.1% (149 of 310), 46.2% (67 of 145), and 49.7% (82 of 165) from all respondents, those trained in teaching hospitals, and those who trained in regional/other-level hospitals respectively. More than 70% raised the issue of practical training for surgical skills, including hands-on training and participation in surgical procedures. Additionally, a lack of supervision or structured curriculum, shortage of infrastructure and personnel, and systematic obstacles were suggested as aspects that required improvement for better-quality training. These trends were comparable between those who were trained in teaching and regional/other-level hospitals.

**Table 3:** Suggestions to improve the quality of surgical rotation (multiple responses)

	Overall (*n* = 149)	Trained at teaching hospitals (*n* = 67)	Trained at regional/other-level hospitals (*n* = 82)	*p*-value	Representative responses
Surgical issues	105 (70.5%)	49 (73.1%)	56 (68.3%)	0.590	
Hands-on or other measures for surgical skill training	67 (45.0%)	31 (46.3%)	36 (43.9%)	0.773	*“More opportunities to learn surgical skills in the theatre and in-house surgical workshops for skills”* *“More opportunities to do cases in theatre under supervision to improve confidence”*
Participation in operating theatre procedures	48 (32.2%)	21 (31.3%)	27 (32.9%)	0.837	*“House officers should be encouraged to assist surgical procedures in theatre and required to perform a minimum number of surgeries like appendicectomies before completing the rotation, which is expected of us once we become medical officers”*
Supervision	27 (18.1%)	10 (14.9%)	17 (20.7%)	0.399	*“Specialist surgeons should improve supervision of house officer training”*
Structured curriculum	22 (14.8%)	10 (14.9%)	12 (14.6%)	> 0.999	*“Everyone should get the equal chance of rotating through the various surgical subspecialties”* *“Define some ‘must know - achieve proficiency’ surgical procedures in theatre”* *“House officers should be involved in the management of the patient at every level. Not just doing reviews and chasing blood and other investigations”*
Infrastructure & resources	13 (8.7%)	7 (10.4%)	6 (7.3%)	0.565	*“Improvement in availability of instruments for surgery and basic surgical procedures”*
Systematic issues	10 (6.7%)	2 (3.0%)	8 (9.8%)	0.186	*“Improved referral systems”*
Personnel	4 (2.7%)	3 (4.5%)	1 (1.2%)	0.327	*“Recruitment of more house officers per facility to ensure equal spread of work burden, in order to give enough time for acquisition of surgical skill in theatre”*
Others	15 (10.1%)	7 (10.4%)	8 (9.8%)		*“More motivation”* *“Preparedness to learn and humility”* *“Less intimidation from senior colleagues”*

Values are n (%)

### Obstacles reported from current MOs on the ground

Among the 125 current MOs, 69.6% (*n* = 87) reported obstacles to medical practice faced on the ground as MOs. The biggest obstacle was their surgical skill (*n* = 32, 36.8%), followed by infrastructure and resources (*n* = 19, 21.8%) and lack of specialist supervision (*n* = 14, 16.1%). MOs working at regional (*n* = 3, 33.3%) or district (*n* = 13, 46.4%) hospitals reported a greater need for improvement of their surgical skills than did those working at teaching hospitals (*n* = 11, 29.7%). However, the perceived need for specialist supervision was comparable among those trained in teaching (*n* = 7, 18.9%), regional (*n* = 2, 22.2%), and district (*n* = 5, 17.9%) hospitals.

## Discussion

The need for improved surgical training and supervision has been highlighted in a previous survey of specialist surgeons who were trained by the GCPS [13]. However, a nationwide enumeration of the operations performed in Ghana demonstrated that 54% of district-level hospitals did not have fully trained surgeons, while 22% of all operations performed were conducted in these hospitals [6]. Therefore, it is crucial to provide high-quality surgical training for HOs and MOs responsible for surgical care in regions where fully trained surgeons are lacking. In the process of surgical housemanship, the modeling (observing experts) and coaching (receiving expert feedback and guidance) steps of cognitive apprenticeship are essential, necessitating the active engagement and involvement of the supervisor. Experiential learning through hands-on experiences also underscores the significance of mentorship and supervised practice, particularly within the operating room, which serves as the primary context for acquiring surgical skills [14]. In our study, limited volume of experience and supervision were the major causes for the lack of confidence in surgical conditions or procedures after completion of surgical housemanship. For MOs already working in underserved areas, supervisory visits and on-site specialist mentorship can facilitate surgical training [13]. However, above all, improving the quality of surgical housemanship is of utmost importance because all MOs undergo this training before being dispatched to work in the districts. The MDC checklist is comprehensive in terms of its content, including essential elements suggested by the Lancet Commission on Global Surgery [1]. Nevertheless, because the learning objectives lack measurable targets, it is difficult for supervisors and trainees to track the trainees’ progress or achievements. Therefore, the MDC’s training objectives need to be practically revised and include achievable and measurable goals, with a minimum number of supervised cases to enhance the commitment of supervisors and increase the exposure of trainees to surgical skills training [7]. Moreover, the massive workload of surgical specialists in LMICs makes it difficult for them to prioritise trainee supervision. A survey of Ghanaian specialist surgeons revealed that although 79% of surgeons were engaged in training or teaching, they spent only 15% of their time on teaching [13]. Appropriate systematic support for supervisors including peer recognition, career development, and incentives should be provided to ensure their focus on education [7, 15].

In the past, patient load in teaching hospitals was larger than that in regional/other-level hospitals due to bypass or impaired quality of services in primary or secondary healthcare facilities [16, 17]. Traditionally, most HOs, MOs, and residents have been placed in teaching hospitals due to the higher concentration of specialists. However, the large contribution of district-level hospitals to the overall surgical volume in Ghana implies decentralisation of the healthcare system [6]. With improvements in the in-country surgeon retention rate and the geographical distribution of specialist surgeons, it is prudent to contemplate a national strategy for the decentralization of services, training, and personnel [13]. For instance, the family medicine training program in Ghana piloted a district-level decentralising specialist training programme [18]. This study revealed that surgical HOs from different hospital levels managed a comparable number of patients, implying that the functional capacity of regional/other-level hospitals has increased to that of teaching hospitals. Moreover, HOs trained in teaching hospitals exhibited lower experience and self-confidence levels in various conditions or procedures compared to their counterparts trained in regional/other-level hospitals This appears to result from the hierarchical structure within teaching hospitals, prioritizing resident training and limiting case exposure for HOs. Teaching hospitals bear the responsibility of providing training, not only for residents but also for HOs, and must ensure that clinical training, assessment, and feedback are not neglected for medical professionals. Considerations for rotations in various subspecialties are essential to establish a structured curriculum that ensures exposure to all necessary fields, including those covered at district-level hospitals. Furthermore, the deployment of surgical trainees, including HOs, MOs, and residents, should be decentralized, with a thoughtful workload and responsibility design to maximize training effectiveness for each cadre.

The cancer burden in Africa is rapidly increasing, with an estimated 1.1 million new cases in 2020 [19]. In Ghana, cancers with the highest incidences are breast and cervical cancers among women and liver and prostate cancers among men [20]. The cancers listed in the MDC checklist accounted for 18.7% (breast), 14.4% (liver), 3.2% (stomach), and 2.6% (colorectal) of all cancers in both sexes in Ghana in 2020 [20]. The experience and self-confidence levels for breast cancer management were the highest among the four cancers, reflecting its high incidence. However, a lack of experience with liver tumours and portal hypertension is concerning, given the relatively higher incidence of liver cancer. In Ghanaian teaching hospitals, the chemotherapy and radiotherapy units are usually excluded from housemanship rotations. As the cancer burden increases, cancer management should not be monopolised by specialists; rather, opportunities should be provided to HOs to participate in cancer diagnosis and treatment. As most HOs are dispatched to rural hospitals once they complete their housemanships, appropriate education and training for HOs in providing differential diagnoses and primary management for patients with cancer in the near future are required to improve early diagnosis, reduced misdiagnosis and overall outcomes of national cancer management.

This study has several strengths and limitations. The results of this study have great implications because they reflect the views of frontline surgical care providers rather than researchers who observe and analyse the situation externally. The study participants underscored their requirement for hands-on surgical skill training and improved supervision during their involvement in surgical procedures, rather than emphasizing challenges identified in policy-level research, such as inadequate infrastructure, resource shortages, personnel deficits, and weak healthcare systems [1, 21, 22]. Additionally, the nationwide scope of this study, focusing on entry-level physicians rather than specialists, is another distinctive feature of this research. However, while there were no significant differences in the number of cases experienced or confidence based on age and gender, it’s important to acknowledge that a higher proportion of men in teaching hospitals compared to regional/other-level hospitals could potentially introduce gender as a confounding factor. The necessity to combine regional and other levels into a single category was driven by limitations in sample size for meaningful comparisons. Nevertheless, it represents another limitation as it involves grouping hospitals from diverse geographic regions and healthcare tiers. Furthermore, it is regrettable that the study could not encompass a more extensive representation of MOs from district-level hospitals.

## Conclusions

Most entry-level physicians are concerned about their surgical skills and request more practical training and better supervision. As surgical housemanship is the only opportunity for formal surgical training for medical graduates, a more structured curriculum with achievable and manageable goals and practical surgical skills training should be established. Considering that regional/other-level hospitals provide a sufficient volume of experience, expanding surgical training in regional/other-level hospitals can alleviate the supervisory burden on teaching hospitals, necessitating the extension of surgeon supervisory roles to regional and district hospitals for nationwide surgical housemanship training.

### Supplementary Information

**Additional file 1: Fig. S1.** Key lessons learned during the surgical rotation. **Table S1.** Details of the evaluation form. **Table S2.** Reasons for not feeling confident in surgical conditions or procedures (multiple responses).

## Data Availability

The datasets used and/or analysed during the current study are available from the corresponding author on reasonable request.
