# Supplementary material for: The need for hands-on training and supervision for entry-level physicians in a country with low surgical staffing density: a nationwide survey in Ghana
Source: BMC Med Educ. 2023 Nov 29;23:904. doi: 10.1186/s12909-023-04880-3 (PMC10687912; doi:10.1186/s12909-023-04880-3)
Supplement: Supplementary file 1 — Additional file 1: Fig. S1. Key lessons learned during the surgical rotation. Table S1. Details of the evaluation form. Table S2. Reasons for not feeling confident in surgical conditions or procedures (multiple responses). [file 12909_2023_4880_MOESM1_ESM.docx]

Original research

**The need for hands-on training and supervision for entry-level physicians in a country with low surgical staffing density: A nationwide survey in Ghana**

Mee Joo Kang, M.D., Ph.D., MScPH, FACS^1,2^, Reuben Kwesi Sakyi Ngissah, M.B. Ch.B., FGCS^1^, Alexis Dun Bo-Ib Buunaaim, M.B., Ch.B., FCS^3^, Richard Baidoo, M.B.B.S., FWACS^4^, Francis Odei-Ansong, M.B., Ch.B., FWACS^5^, Theodore Wordui, M.B., Ch.B.^6^, Ernest Kwame Adjepong-Tandoh, M.B., Ch.B.^6^, Paa Kwesi Baidoo, M.B., Ch.B., FWACS, FGCS^7^, James Emmanuel Kwegyir Aggrey-Orleans, M.B., Ch.B., FWACS^8^

^1^ Department of Surgery, Greater Accra Regional Hospital, Accra, Republic of Ghana

^2^ Department of Surgery, National Cancer Center, Goyang, Republic of Korea

^3^ Department of Surgery, Tamale Teaching Hospital, Tamale, Republic of Ghana

^4^ Department of Surgery, Cape Coast Teaching Hospital, Cape Coast, Republic of Ghana

^5^ Department of Surgery, Tema General Hospital, Tema, Republic of Ghana

^6^ Department of Surgery, Korle-Bu Teaching Hospital, Accra, Republic of Ghana

^7^ Department of Surgery, Komfo-Anokye Teaching Hospital, Kumasi, Republic of Ghana

^8^ Department of Surgery, 37 Military Hospital, Accra, Republic of Ghana

**Corresponding author**: Reuben Kwesi Sakyi Ngissah, M.B. Ch.B., FGCS

Department of Surgery, Greater Accra Regional Hospital

P.O.Box 473, Accra, Republic of Ghana

Tel: +233-20-630-0921

E-mail: [rngissah@yahoo.com](mailto:rngissah@yahoo.com)

ORCID ID : 0000-0003-3801-7978

**Supplementary Figures and Tables**

Fig. S1. Key lessons learned during the surgical rotation.

**
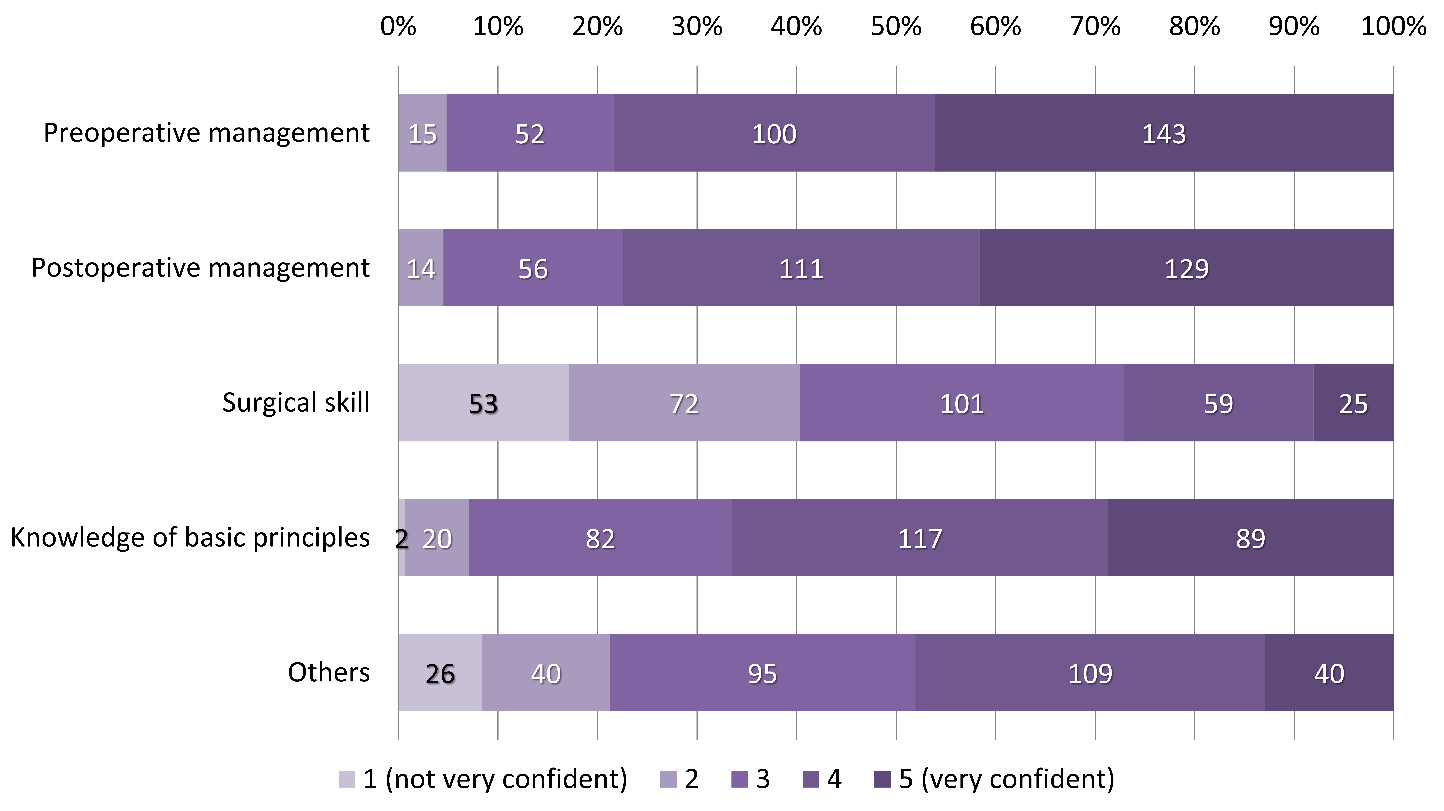
**

Table S1. Details of the evaluation form.

| Questions | Options and measures |
| --- | --- |
| **Demographics** |  |
| Age group, gender * | ≤25, 26–30, 31–35, 36–40, ≥40 years old  Male, female, decline to respond  Finished housemanship |
| Which stage of medical training are you in? * | House Officer, completed surgical rotation  Completed housemanship, yet to work as a Medical Officer  Medical Officer |
| In which hospital did you have your surgical rotation? * | Korle-Bu Teaching Hospital  37 Military Hospital  Komfo-Anokye Teaching Hospital  Cape Coast Teaching Hospital  Tamale Teaching Hospital  Greater Accra Regional Hospital  Others |
| When have you completed your surgical housemanship? * | Free text |
| In which discipline are you currently working as a medical officer? * | Surgery related disciplines  Non-surgical disciplines  Both surgical and non-surgical disciplines  Not a medical officer |
| What is the level of hospital where you are working as a medical officer? * | Teaching hospitals  Regional hospitals  District hospitals  Not a medical officer  Other (free text) |
| **MDC checklist (35 conditions or procedures)** |  |
| How many cases have you experienced the following conditions/procedures? * | Never  1~5 cases  6~10 cases  11~20 cases  More than 21 cases |
| Are there any conditions/procedures you think not appropriate for surgical housemanship? * | Yes  No |
| If yes, please name them and briefly explain the reason. | Free text |
| Are there any conditions/procedures you want to add for the surgical housemanship curriculum? | Free text |
| In what extent do you feel confident in managing the following conditions/procedures if you were on your own after completing surgical housemanship? * | 1 (not very confident) ~ 5 (very confident) in 5-point Likert-type scale |
| For the conditions/procedures that you don’t feel confident, please explain the reason. * | Multiple selection for;  Limited number of patients  Limited supervision  Limited resources and infrastructure  Limited timeframe for surgical rotation  Not interested in surgical conditions  Other (free text) |
| **Level of satisfaction and suggestions** |  |
| In overall, how satisfied were you with the surgical housemanship? * | 1 (not very satisfied) ~ 5 (very satisfied) in 5-point Likert-type scale |
| How relevant and helpful do you think the surgical rotation was with your work after housemanship? * | 1 (not very relevant) ~ 5 (very relevant) in 5-point Likert-type scale |
| What were your key take aways from surgical rotation? * | 1 (not very confident) ~ 5 (very confident) in 5-point Likert-type scale for the following subdomains;  Preoperative management  Postoperative management  Surgical skills  Knowledge of basic principles  Others |
| What were main obstacles you’ve faced working as a medical officer, in relation to surgical cases? | Free text |
| Please describe any suggestions to improve the quality of the surgical housemanship which might have helped you more working as a medical officer | Free text |

* Compulsory questions

Table S2. Reasons for not feeling confident in surgical conditions or procedures (multiple responses).

| Theme | N=638 (%) |
| --- | --- |
| Limited number of patients | 237 (76.5%) |
| Limited resources and infrastructure | 141 (45.5%) |
| Limited supervision | 118 (38.1%) |
| Limited timeframe for surgical rotation | 93 (30.0%) |
| Not interested in surgical conditions | 18 (5.8%) |
| Others | 31 (10.0%) |

*Values are n (%).
